# 

**Published:** 1851-06

**Authors:** 


### WESTERN JOURNAL

OF

### MEDICINE AND SURGERY.

EDITORS.

---

LUNSFORD P. YANDELL, M. D.
PROFESSOR OF PHYSIOLOGY AND PATHOLOGICAL ANATOMY IN THE UNIVERSITY OF LOUISVILLE.

AND

THEODORE S. BELL, M.D.

### INDEX TO VOL. VII.

[THIRD SERIES.]

A

Abortion habitual, savine in the treatment of...................246
A case of Tetanus...............105
of Traumatic Tetanus....112
of Urinary Calculi..........359
Acetate of Potash in some affections of the skin...................... 90
Acetate of Potash in gonorrhea....145
Acetate of potash, rheumatic fever treated by ........................546
Acephalous monster................212
Acid, nitro-muriatic bath...........230
Action of mercurial ointment, and vapor of mercury................ 87
Adjuster Jarvis's....................176
Alabama State medical association..367
Alimentary substances absorption of.450
Alterative medicines Chipley on.... 39
American medical association......184
An anomalous case of horny excrescences, by A. Evans M. D......369
Anatomical lucubrations at the Cape of Good Hope.............152
Anthelmintic the ....................146
Anti-lithic paste.................... 92
Antiseptic, chloroform as an.......176
Ascites ...............................175
Auvert's Plates........................180

B

Banksia Abyssinica..................146
Bartlett Professor....................459
Bath, nitro-muriatic and uses of....230
Bell on Small Pox, vaccination &c. 22
Bell's case of Tetanus..............112
of Convulsions ........ 116
Bell on epidemic influences.........277
on Practical medicine..........384
Bell's report of poisoning with sulphuretted hydrogen gas.........409
Belladonna tincture of in the treatment of tetanus.................207
Buttons in the intestines...........393

C

Caffeine citrate of in hemicrania...152
Camphor, odor of musk destroyed by.................................... 88
Cancer, efficacy of low temperature.154
Cape of Good Hope anatomical lucubrations ......................... 153
Carbonagogue medication, Rogers Prof. on..........................461
Carbonagogue medication, editorial.548
Caries of bone, treatment of abscesses connected with..........533
Card, Prof. Eve's.................... 92
Cardiac dropsy, treatment of.......134
Case of urinary calculi, by Professor Dugas...........................359
Case of poisoning with sulphuretted hydrogen gas...................409
Catalogue of inorganic substances employed in medicine............ 11
Cervix and os uteri rigidity of.......177
Chipley on alterative medicines.... 93
Chloroform remarkable effects of...116
Chloroform as an anti-septic........175
Citrate of caffeine, in hemicrania..152
Clinical observations in private practice...................................185
Clinical lecture on kousso.........242
Cobb's case of facial palsy......... 19
Cobb's translations............191—371
" Case of tetanus............105
Cod liver oil in phthisis........... 88
" " "............344
Comparative liability of males and females to insanity..............209
Comparative curableness of insanity in the sexes.......................209
Comparative value of the diagnostic signs of pneumonia..............214
Compression, popliteal aneurism successfully treated by .........529
Compression, femoral aneurism cured by.........................527
Constipation habitual, treatment of.228
Continuation of the case of facial palsy, reported for this Journal, December, 1849................ 19
Convulsions, remarkable effects of chloroform in.....................116

D

Davis's history of medicine, et cet..175
Death from heart disease in a bath..150
Demonstrative midwifery...........275
Dengue fever...................... 81
Diagnosis of tape worm...........141
" errors in...........211
Diagnostic signs of pneumonia.....214
Diagnosis differential of gastralgia, and other more serious affections of the stomach.....................217
Differential diagnosis of gastralgia and other affections of the stomach................................. 217

Dieuretic, water mellon seed as a... 19
Diagnostic value of epigastric pains. 219
Disorders, strumous, treatment of with walnut leaves ............ 264
Dislocation of femur, reduced on the sixty-eighth day by Jarvis's adjuster ....................... 235
Doses large of quinine, in neuralgia 148
Double ovarian dropsy ............ 397
Dowler on the necropolis of New Orleans ........................ 168
Drake B. P., on inverted toe-nail.. 104
Dropsy cardiac ................... 134
" ovarian double ............ 397
Dugas's extraordinary case of urinary calculi ................... 359
Dumb Bell, urinary deposits ....... 484

E

Efficacy of a low temperature in cancer ......................... 154
Eighth general meeting of Sydenham society ........................ 161
Epidemic influences .............. 277
Epigastric pains, diagnostic value of 219
Epilepsy with spectral illusions.... 129
Epilepsy, Surgical treatment of.... 85
Errors in diagnosis ............... 211
Ethnology ........................ 265
Evan's case of horny excrescences. 371
Eve Prof P. F. ................... 179
Eve's Prof. card ................. 92
Excrescences horny ............... 369
Expulsion of tape-worm ........... 152

F

Febrile paroxysm, quinine in..... 198
Femur dislocation of, reduced on the sixty-eighth day by Jarvis's adjuster ....................... 235
Femoral aneurism cured by compression ....................... 527
Ferri iodidi syrup ................ 89
Fever dengue ..................... 82
Fever puerperal, treatment of..... 247
France, hare lip in .............. 208
Frick on renal affections ......... 77

G

Gastralgia, differential diagnosis between it aud other affections of the stomach ..................... 217
Geological theory of cholera examined ........................... 334
Gonorrhea treated with acetate of potash .......................... 145
Glycerine, Wakely on ............. 405

H

Habitual constipation, treatment of, 228
Harelip in France ................ 208
Haskin's clinical observations in private practice ............. 1, 185
Hemicrania, citrate of caffeine in.. 125
Hemorrhoids, linseed oil in ....... 88
Headache cured by full inspirations ........................... 210
Heart disease, death from ........ 150
Hemp, Indian, as an oxytoxic..... 256
History of Medicine, etc.......... 175
Horny excrescence, case of ....... 369
Hot water in sprains, Jackson on.. 395
Hydrocele ........................ 175
Hydrogen, sulphuretted gas, poisoning by ........................ 409

I

Illusions, spectral, in epilepsy..... 129
Indian hemp as an oxytoxic ....... 256
Infants, nourishment for ......... 132
Influences, epidemic ............. 277
Injuries of nerves ............... 385
Insanity, statistics of, comparative liability of males and females.... 209
Insanity, comparative curableness in the sexes ..................... 209
Inspirations, full, for the cure of sick headache ................. 210
Inverted toe-nail, new remedy for.. 104
Iodognosis. ...................... 451
Itch, treatment of ............... 526

J

Jackson on hot water in sprains... 395
Japan remedy for sterility ........ 255
Jarvis's adjuster ............. 182; 235

K

Kentucky surgery ................ 403
Knox on races of men ............ 79
Kousso .......................... 146
success of, in the expulsion of tape worm ............ 152
a new anthelmintic..... 236,242

L

Lacteals, functions of ........... 450
Lea's geological theory examined ........................... 336
Linseed oil in hemrrhoids......... 88
Lithotrity, extraordinary success of Professor Dugas ................ 359

M

Meetings of learned bodies ....... 366
Medical association, American.... 184
Medical Society of London ....... 534
Medicated soaps for skin disease.. 217
Medication carbonagogue, Rogers on ............................ 461
Meigs, Prof., on women and children ........................... 77
Microscope, an improved one..... 546
Midwifery, demonstrative ........ 275
Monster, acephalous ............. 212
Morbid poisons .................. 220
Musk, odor of, destroyed by camphor ........................... 88
Nashville Journal of Medicine and Surgery ........................ 368

N
Nerves, injury of....................385
Neuralgia, large doses of quinine in.148
New Orleans Necropolis of........168
Nitro-muriatic acid bath...........230
Nourishment for infants...........132
O
Obituary.............................460
Obstructions, symptoms of from buttons in the intestines...............393
Ovarian dropsy double............397
Oxytoxic, Indian hemp as an......256
P
Pains epigastric, diagnostic value of219
Palpitation symptomatological value of...................................232
Palsy facial..........................319
Paste anti-lithic..................... 92
Pains, treatment of veneral diseases in......................................120
Paroxysm febrile, quinine..........198
Pharmacy of the Greeks and Romans...................................371
Pathological Society of London...538
Phthisis pulmonalis, cod liver oil in.344
Phthisis, Cod liver oil in........... 88
Plates Auvert's.......................180
Pneumonia, diagnostic signs of....214
Poisons morbid......................220
Potash acetate of, in some diseases of the skin......................... 90
Potash acetate of, in gonorrhea....145
Practical illustrations of the efficacy of a low temperature in cancer..154
Practical medicine..................384
Proceedings of the medical association of Alabama....................367
Prof. P F. Eve.......................179
Puerperal fever, treatment of.......246
Q
Quinine large doses of in neuralgia 148
" in febrile paroxysm.......198
R
Renal affections..................... 77
Renal diseases, value of a chemical and microscopical examination of the urine as a means of diagnosis in.......................................523
Report of sanitary committee of Massachusetts.......................458
Researches upon the necropolis of New Orleans.......................168
Rheumatic fever treated by acetate of potash...........................540
Rochester rappings................. 157
Rogers Prof. on carbonagogue medication.................................461
S
Savine in the treatment of habitual abortion..............................246
Sck head ache cured...............210
Silliman Professor............480—538
Silliman's Journal.................. 80
Sinapisms..............................458
Skin, diseases of, treated by acetate of potash.......................... 90
Skin diseases of, treated by medicated soaps.........................217
Small pox, vaccination &c......... 22
Small-pox, vaccination as a preventive of...............................524
Smith's microscope..................546
Soaps medicated.....................217
Speculum uteri.......................173
Sprains, hot water for..............395
Sterility, a Japanese remedy for...255
Stethoscope the, a medical journal.368
Stomach diseases of, diagnosis....217
St. Bartholomew's Hospital, results of the use of chloroform in 9000 cases at........................520
Strumous diseases, treated with walnut leaves..........................204
Symptomatological value of palpitation.....................................232
Syrup iodide ferri.................. 89
T
Tape worm diagnosis of...........141
" " expelled by koussi....152
Temperature low, in treatment of cancer..................................154
Tetanus with recovery.............175
Tetanus traumatic, treated with tincture belladonna................207
The races of men................... 79
The odor of musk destroyed by Camphor........................... 88
The speculum uteri.................173
The history of medical education..464
Therapeutics, agency of in relieving rigidity of os and cervix uteri....177
Translations from the French of M. Cap, by W. H. Cobb, M D. 191, 510
Transactions of Ohio state medical society................................459
Traumatic tetanus cured with belladonna..................................207
Treatment of cardiac dropsy.......134
Treatment of habitual constipation.228
" " " abortion....246
Treatment of puerperal fever.......237
Treatment of Itch....................526
Treatment of abscesses connected with caries of bone...............533
U
University of Louisville......364, 345
Urinary calculi, Dugas's wonderful case of..................................359
Urinary deposits.....................384
Uteri speculum.......................173
Uteri rigidity of os and cervix, treament of........................177

V

Vacaination, re-vaccination, variola &c....456

Vaccination a preventive of small-pox....524

Veneral diseases treatment of in Paris....120

W

Walnntleaves in strumous disorders264

Warren's Dr. address....366

Water hot, for sprains....395

Water mellon seed as a diuretic.... 91

Woman, her diseases and remedies. 77

Y

Yandell Dr. D. W. on cod liver oil, in phthisis pulmonalis....344

REVIEWS.

Woman, her diseases and remedies. A series of letters to his class, by Charles D. Meigs, M. D., Professor of Midwifery, and the diseases of women and children, in the Jefferson Medical College at Philadelphia, &c. &c.... 77

Renal Affections: their diagnosis and pathology. By Charles Frick, M. D.... 77

The Races of Men: A Fragment. By Robert Knox, M. D.... 80

Report of the Eighth General Meeting of the Sydenham Society, held at the Society's Rooms, on Wednesday, May 1st, 1850.....161

Researches upon the Necropolis of New Orleans, with brief allusions to its Vital Arithmetic. By Bennet Dowler, M.D., of New Orleans.....168

History of the Strange Sounds or Rappings, heard in Rochester and Western New York, and usually called the Mysterious Noises, &c. By D. M. Dewey, Rochester 1850.

Exposition of the Rochester Knockings. By Austin Flint, M. D., Charlas Lee, M. D., and C. B. Coventry, M. D.....257

United States Exploring Expedition, during the years 1838, 1839, 1840, 1841, 1842, under the command of Charles Wilkes, U. S. N. Vol ix.....265

A Practical Treatise on the most common Diseases of the South, exhibiting their peculiar nature, and the corresponding adaptation of Treatment. By Thompson McGown, M. D.....415

Ether and Chloroform: their employment in Surgery, Dentistry, Midwifery, Therapeutics, etc. By J. B. Flagg, M. D.....418

The Medical Student's Guide in Extracting Teeth; with numerous cases in the surgical branch of Dentistry. With illustrations By S. S. Hornor.....430

First Principles of Medicine. By Archibald Billing, M. D., A. M., F. R. S.....430

Review of Chemistry for Students, Adapted to the courses as taught in the principal Medical Schools in the United States. By John G. Murphy, M. D.....433

The American Journal of Science and the Arts. Conducted by Professors B. Silliman, B. Silliman, Jr., and James D. Dana, aided in the Departments of Chemistry and Physics by Dr. Wolcott Gibbs.....435

Cases of Vesico Vaginal Fistula. By George Hayward, M. D.... 437

An Address, delivered before the Suffolk District Medical Society, at its second anniversary meeting, Boston, March, 1851. By Sam'l Parkman, M.D., M.M, S.S......439

An Address to the Graduates of the Medical Department of the St. Louis University. By A. Litton, M.D.... 441

Valedictory Address before the Graduates of Washington University, Baltimore. By Professor T. E. Bond.....441

Valedictory Address to the Graduates of the Medical Department of the University of Pensylvania. By W. E. Horner, M. D......441

The progress of Medicine in America. By Horace Green, M. D..442

Half-yearly Abstract of the Medical Sciences. Edited by W. H. Ranking, M. D., Cantab., assisted by W. W. A. Guy, M. D., George Day, M. D., Henry Ancell, M.D. and W. Kirkes, M. D.....446

Annual Report of the Louisville Marine Hospital.....447

Report of the Diseases and their results, coincident with Admission and Discharges at the Louisville Marine Hospital, from March 1st, 1850, to March 1st, 1851.....448

### *Receipts of Medical Journal for May*, 1851.

| | | |
|---|---|---|
| Dr. John E. Shipp, Centreville, Tenn., | - - - | $5 00 |
| J. C. Whitridge, Lebanon, Ohio, | - - - | 5 00 |
| W. E. Whitson, Victory, Tenn., | - - - | 5 00 |
| Mastic & O'Neal, Bath, Ill., | - - - - | 5 00 |
| —— Brookhart, Bardstown, Ky., | - - - | 5 00 |
| —— Lister, Office, | - - - - - | 5 00 |

The Western Journal of Medicine and Surgery is published monthly by the undersigned, at the corner of Main and Fifth streets, Louisville, at $5 per annum, payable in advance. Each number contains 96 pages, making two volumes in the year of more than 550 pages each.

Contributions to its pages by the Physicians of the Valley of the Mississippi, addressed to the Editors, are respectfully solicited.

Letters on business, to be addressed, postage paid, to the publishers.

PRENTICE & CO.

